# Chronic Fluoxetine Treatment Desensitizes Serotoninergic Inhibition of GABAergic Inputs and Intrinsic Excitability of Dorsal Raphe Serotonin Neurons

**DOI:** 10.3390/brainsci15040384

**Published:** 2025-04-08

**Authors:** Wei Zhang, Ying Jin, Fu-Ming Zhou

**Affiliations:** 1 Department of Pharmacology, Hebei University of Chinese Medicine, Shijiazhuang 050200, China; zhangwei@hebcm.edu.cn; 2 Department of Pharmacology, University of Tennessee College of Medicine, Memphis, TN 38163, USA; jin_ying@fudan.edu.cn; 3 Institute of Science and Technology, Fudan University, Shanghai 200433, China

**Keywords:** 5-HT autoinhibition, antidepressant, dorsal raphe, G-protein-activated inwardly rectifying K (GirK) channel, neuroplasticity, selective serotonin reuptake inhibitor

## Abstract

**Background:** Dorsal raphe serotonin (5-hydroxytryptamine, 5-HT) neurons are spontaneously active and release 5-HT that is critical for normal brain function and regulates mood and emotion. Serotonin reuptake inhibitors (SSRIs) increase the synaptic and extracellular 5-HT level and are effective in treating depression. Treatment of two weeks or longer is often required for SSRIs to produce clinical benefits. The cellular mechanism underlying this delay is not fully understood. **Methods and Results**: Using whole-cell patch clamp recording in brain slices, here we show that the GABAergic inputs inhibit the spike firing of raphe 5-HT neurons. This GABAergic regulation was reduced by 5-HT; additionally, this 5-HT effect was prevented by the G-protein-activated inwardly rectifying potassium (GirK) channel inhibitor tertiapin-Q, indicating a contribution of 5-HT activation of GirK channels in GABAergic presynaptic axon terminals. Equally important, after 14 days of treatment with fluoxetine, a widely used SSRI type antidepressant, the 5-HT inhibition of GABAergic inputs was downregulated. Furthermore, chronic fluoxetine treatment downregulated the 5-HT activation of the inhibitory GirK current in 5-HT neurons. **Conclusions:** Taken together, our results suggest that chronic fluoxetine treatment, by blocking 5-HT reuptake and hence increasing the extracellular 5-HT level, can downregulate the function of 5-HT1B receptors on the GABAergic afferent axon terminals synapsing onto 5-HT neurons, allowing extrinsic GABAergic neurons to more effectively influence 5-HT neurons; simultaneously, chronic fluoxetine treatment also downregulated somatic 5-HT autoreceptor-activated GirK channel-mediated hyperpolarization and decrease in input resistance, rendering 5-HT neurons resistant to autoinhibition and leading to increased 5-HT neuron activity. These neuroplastic changes in raphe 5-HT neurons and their GABAergic afferents may contribute to the behavioral effect of SSRIs.

## 1. Introduction

The serotonin (5-hydroxytryptamine, 5-HT) neurons in the dorsal raphe nucleus (DRN) project to every forebrain area in rodents and primates including humans [1,2,3,4,5,6,7]. 5-HT, the key neurotransmitter of these neurons, contributes critically to the development and synaptic connectivity of these brain areas [8,9,10,11,12]. 5-HT is also important for the normal operation and cognition of the adult brain, as indicated by the fact that 5-HT agonists such as lysergic acid diethylamide (LSD) can alter human cognition and trigger powerful hallucinations [13,14,15]. Further supporting the importance of the 5-HT system, selective serotonin reuptake inhibitors (SSRIs) that increases the extracellular 5-HT level are effective for treating depression symptoms in humans, at least in sub-groups of patients [16,17]. Thus, controlling and regulating 5-HT neuron spiking activity that trigger 5-HT release in brain target areas is important for normal brain function and may contribute to the pathogenesis of depression and other neuropsychiatric-behavioral disorders.

5-HT neurons often fire around 3 Hz in a quiet awake state [18,19,20,21,22]. This tonic firing is altered when the animal’s cognitive and behavioral state and its environment are changed. Synaptic inputs likely contribute to the behavior-related changes in 5-HT neuron firing. Studies have indicated that GABAergic inputs inhibit raphe 5-HT neuron firing [23,24]. For example, inhibition of 5-HT neuron spike firing or decreased 5-HT release affects sleep [21,25,26,27,28,29,30].

This inhibition of 5-HT neuron activity may be mediated, at least partially, by GABAergic input from extrinsic sources such as the hypothalamus, lateral habenula nucleus, mesopontine rostromedial tegmental nucleus (RMTg), lateral preoptic area and the pontine ventral periaqueductal gray including the DRN, ventral tegmental area (VTA), and substantia nigra pars reticulata (SNr) [31,32,33,34,35,36,37,38,39,40,41]. These GABAergic inputs can affect the spike firing of the raphe 5-HT neurons. Further, GABAergic afferents to 5-HT neurons may express 5-HT1B receptors that may inhibit GABA release, providing another mechanism that regulates 5-HT neuron activity [42].

Raphe 5-HT neurons can also inhibit themselves (autoinhibition) by releasing 5-HT in the raphe and expressing inhibitory 5-HT1A autoreceptors [43,44,45,46,47]. 5-HT1A receptor activation triggers the opening of G-protein-activated inwardly rectifying K (GirK) channels, leading to hyperpolarization and inhibition of these neurons [18,22,48,49,50,51,52,53]. Furthermore, chronic (≥2 weeks) fluoxetine treatment, matching the required duration for clinical antidepressant effects to appear, has been shown to downregulate or desensitize 5-HT1A receptors [45,54,55,56] and remove the initial 5-HT neuron firing inhibition after selective 5-HT reuptake inhibitor treatment (detected by in vivo extracellular spike recording) [57,58,59,60,61,62]. This 5-HT1A receptor neuroadaptive downregulation has been suggested to be critical to fluoxetine’s antidepressant effect [16,17,63]. Indeed, molecular genetics-mediated inactivation of or reduction in these 5-HT1A autoreceptors had antidepressant-like effects [64,65]. These previous studies used biochemical methods and extracellular recording methods. Thus, a knowledge gap exists about (1) the potential effects of chronic fluoxetine on 5-HT-activated GirK-mediated hyperpolarization and the associated changes in intrinsic excitability in dorsal raphe 5-HT neurons, and (2) potential changes in 5-HT regulation of GABAergic inputs to these 5-HT neurons. In our study, we sought to bridge this knowledge gap by chronically treating mice with fluoxetine and then performing whole-cell patch-clamp recording in raphe 5-HT neurons.

## 2. Materials and Methods

### 2.1. Animals

All experimental protocols were approved by the Institutional Animal Care and Use Committee at the University of Tennessee Health Science Center, Memphis, Tennessee (animal protocol #17-033.0). Normal wild-type C57Black/6J breeder mice were purchased from The Jackson Laboratory and offspring mice were used for experiments. No transgenic were used in this study. Mice had free access to food and water. The room light was on from 7:00 a.m. to 7:00 p.m. and off for the night. Male and female mice (starting on PN 13 days) were administered intraperitoneally the antidepressant fluoxetine (10 mg/kg) or saline for two weeks (once daily). The 2-week duration was mainly based on the published results of Blier & de Montigny (1983) [57] and Czachura & Rasmussen (2000) [61]. Fluoxetine was chosen because it is a commonly prescribed and hence important antidepressant. The fluoxetine dose (10 mg/kg) was based on the mouse and rat fluoxetine dose-plasma concentration data and in vivo raphe 5-HT neuron spiking data of Czachura & Rasmussen (2000) [61], Ansorge et al. (2008) [66] and Sawyer (2011) [67]. The required numbers of mice for sufficient statistical power for our quantitative experiments were estimated by power analysis performed using a power analysis calculator provided by Boston University.

### 2.2. Brain Slice Preparation

Male and female mice (PN27-30) were rapidly decapitated and their brains were quickly dissected. The brainstem was rapidly removed and placed in ice-cold, high-sucrose, artificial cerebral spinal fluid (ACSF) that contained the following (in mM): 220 sucrose, 2.5 KCl, 1.25 NaH_2_PO_4_, 2 ascorbic acid, 25 NaH_2_CO_3_, 0.5 CaCl_2_, 7 MgCl_2_, and 20 D-glucose, pH 7.4 (continuously bubbled with 95% O_2_–5% CO_2_). Three to four coronal midbrain slices containing the dorsal raphe nucleus were cut (300 μm thick) using a Leica vibratome (VT1200S, Leica Microsystems, Wetzlar, Germany) and immediately transferred to a holding chamber filled with the normal extracellular solution (in mM): 2 ascorbic acid, 125 NaCl, 2.5 KCl, 1.25 NaH_2_PO_4_, 25 NaH_2_CO_3_, 2.5 CaCl_2_, 1.3 MgCl_2_, and 10 D-glucose, pH 7.4 (when continuously bubbled with 95% O_2_–5% CO_2_). This extracellular solution was also the normal perfusing solution. After incubation at 34 °C for 30 min, the slices were maintained at room temperature (22–24 °C) until being transferred to the recording chamber on the stage of the microscope. Drugs were applied via the perfusing solution. The perfusion rate was 2 mL/min.

### 2.3. Electrophysiological Recording

**Figure 1:**
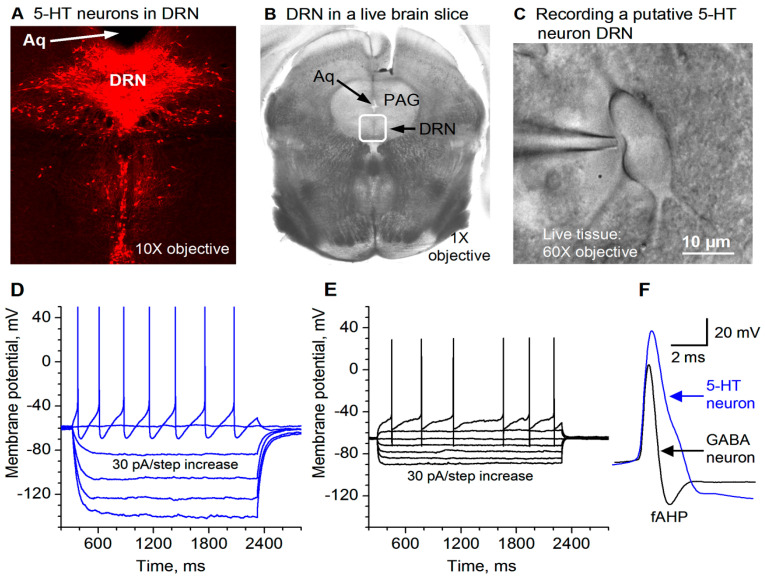
Recording and identification of 5-HT neurons in the dorsal raphe nucleus (DRN) in mouse brain slices. (**A**) Tryptophan hydoxylase (TPH) immunostaining identifies 5-HT neurons in mouse DRN. Aq: aqueduct. (**B**) A live coronal brain slice containing the DRN viewed under a low-power objective. The boxed area is roughly the DRN. Aq: aqueduct. PAG: periaqueductal gray. (**C**) A putative 5-HT neuron in the DRN being patch-clamped. (**D**) Membrane potential responses to current injection into a putative 5-HT neuron in the DRN. The current strength started at −120 pA and the step size was 30 pA. For display purposes, the 5-HT neuron spike amplitudes were truncated by ~5 mV (the peak reached ~ +55 mV in this neuron). (**E**) Membrane potential responses to current injection into a putative GABA neuron in the DRN. The current strength started at −120 pA and the step size was 30 pA. (**F**) A high temporal resolution display showing that the action potential duration and amplitude for DRN 5-HT neurons are clearly longer and larger than those for DRN GABA neurons.

Conventional whole-cell patch-clamp recordings were performed in the recording chamber maintained at 30 °C by a solution heater. As illustrated in Figure 1A,B, the dorsal raphe neurons were visualized in the midline region ventral to the aqueduct, using an Olympus upright microscope (BX51WI) fitted with a 60x water immersion lens and DIC optics and a Zeiss Axiocam MRm digital camera. The slice was submerged in and perfused with a normal extracellular solution equilibrated with 95% O_2_–5% CO_2_. Whole-cell recording pipettes were pulled from borosilicate glass capillary tubing (KG-33, 1.65 mm outer diameter, 1.10 mm inner diameter, King Precision Glass, Claremont, CA, USA) on a two-stage PC-10 micropipette puller (Narishige, Tokyo, Japan).

Cells were recorded in whole cell configuration in voltage- and/or current-clamp mode. Recordings were made with an Axopatch 200B patch-clamp amplifier (Axon Instruments, USA) and pCLAMP 9.2 software (Clampex for data acquisition and Clampfit for data analysis). Signals were filtered at 5 kHz, digitized at 10 kHz with the Digidiata 1322A interface (Axon Instruments), and stored in a computer hard disk for off-line analysis using Clampfit.

**Figure 2:**
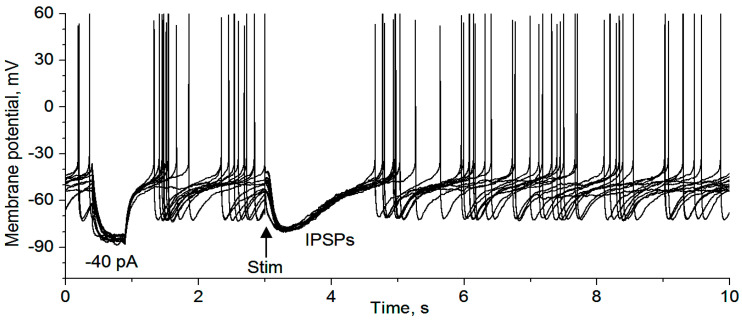
Qualitative demonstration that IPSPs inhibit raphe 5-HT neuron action potential firing. The recording was made with a KSO_3_CH_4_-based intracellular solution (for producing hyperpolarizing IPSPs) in a saline-treated mouse. The first hyperpolarization pulse was induced by a −40 pA pulse to monitor input resistance and to provide an estimate of the strength of the synaptic input underlying the IPSPs: for this the 5-HT neurons, the synaptic current was less than 40 pA. Ten sweeps are displayed here. Stim, local electrical stimulation (20 μA 0.1 ms, delivered every 30 s).

A potassium methanesulfonate (KSO_3_CH_3_)-based low Cl^-^ intracellular solution (in mM: 135 KSO_3_CH_3_, 5 KCl, 0.5 EGTA, 10 HEPES, 2 Mg-ATP, 0.2 Na-GTP, and 4 Na_2_-phosphocreatine, pH 7.25, 280–290 mOsm) was used for recording the natural hyperpolarizing GABA_A_ inhibitory postsynaptic potentials (IPSPs) and their effects on spiking activity (Figure 2). A high Cl^-^ intracellular solution (in mM: 135 KCl, 0.5 EGTA, 10 HEPES, 2 Mg-ATP, 0.2 Na-GTP, and 4 Na_2_-phosphocreatine, pH 7.25, 285 mOsm) was used for recording inward GABA_A_ inhibitory postsynaptic currents (IPSCs) at –70 mV. The patch pipette resistance was about 2 MΩ. Access resistance change was <15% during recording. Liquid junction potentials were not corrected.

Evoked IPSCs (eIPSC) were electrically stimulated via a bipolar stimulating electrode; stimulus timing and intensity were controlled by using a Master-8 pulse generator (A.M.P.I.) and an A360 stimulus isolator (World Precision Instruments, Sarasota, FL, USA) in the presence of ionotropic glutamate receptor blockers 6-cyano-7-nitroquinoxaline-2,3-dione (CNQX, 10 μM, to block non-NMDA glutamate receptors) and D-2-amino-5-phosphonopetanoic acid (D-APV, 20 μM, to block NMDA receptors). The dorsal raphe neuron under recording was usually 100 μm away from the two tips of the stimulating electrode. Stimulus intervals were 30 s unless otherwise stated. Input resistance was measured in the whole cell configuration in current-clamp mode by injecting a current pulse of –20 pA with a duration of 1000 ms.

### 2.4. Statistics

Each mouse served as one replicate or data point: we either recorded one 5-HT neuron from one mouse, or averaged the data from one mouse when Two or Three neurons were recorded in the mouse (counted the average as one neuron). The data are presented as the mean ± SEM. The Kolmogorov–Smirnov test (K-S test), one-way ANOVA with post-hoc Tukey’s test and an unpaired t-test were used for statistical comparison. *p* < 0.05 was used as one indicator of significant difference; we are aware that the overall data provide important indications if there are significant differences between experimental groups [68,69]. Calculations were performed using StatMost software (version 3.6) (Dataxiom Inc., Los Angeles, CA, USA).

### 2.5. Drugs

Picrotoxin, DL-2-amino-5-phosphonopentanoic acid (AP-5), 6-cyano-7-nitroquinoxaline-2,3-dione (CNQX), 5-HT, tetrodotoxin (TTX), and tertiapin-Q were purchased from Tocris or Sigma. Fluoxetine was either purchased from Tocris or supplied by the NIMH Drug Supply program. 5-HT was purchased from Sigma, prepared as a 10 mM stock solution in pure water, and stored at −20 °C and was used at the final concentration of 10 μM.

### 2.6. DRN 5-HT Neuron Mapping with Tryptophan Hydroxylase (TPH) Immunostaining

To guide our electrophysiological recording, we also immunostained the tissue sections for TPH, a key 5-HT synthesis enzyme, to visualize DRN 5-HT neurons (Figure 1A). We used conventional immunohistochemical procedures that we have published [70,71]. Briefly, free-floating sections (50 μm in thickness) were incubated with 2% fat-free milk, 1% bovine serum albumin (BSA), and 0.8% Triton X-100 in 0.01 M PBS for 1 h at room temperature to block nonspecific staining. Then, these free-floating tissue sections were incubated with a primary mouse anti-TPH antibody (diluted 1:500, purchased from Sigma, Cat. # MAB5278) for 48 h at 4 °C. After 3 × 10 min PBS rinses to wash out excess primary antibody, the tissue sections were incubated with Alexa Fluor 568 (red) donkey anti-mouse secondary antibody (diluted 1:400, purchased from Invitrogen) for 2 h at room temperature, followed by rinsing 3 × 10 min, and being cover-slipped and digitally photographed on a fluorescence microscope.

## 3. Results

### 3.1. Electrophysiological Identification of Dorsal Raphe 5-HT Neurons

Under visual guidance of a video-microscope equipped with a 60x water immersion lens and DIC optics, we made whole-cell patch clamp-recordings in neurons in the dorsal raphe of mice. To record putative 5-HT neurons, we targeted relatively large neurons in the DRN (~20–25 μm in the longest dimension, Figure 1B,C) because literature data indicate that large neurons in the dorsal raphe are 5-HT neurons. Our presumed 5-HT neurons (5-HT neurons hereafter) had the following basic electrophysiological properties: a resting membrane potential of –61.3 ± 1.2 mV, a high spike peak amplitude of 93.3 ± 1.3 mV, a wide action potential duration of 2.94 ± 0.11 m at the spike base and a whole-cell input resistance of 362.2 ± 5.4 MΩ (n = 18). Although not our focus in this study, for the purpose of comparison, we also recorded presumed GABAergic neurons by targeting smaller neurons (≤15 μm in the longest dimension). As shown in Figure 1D–F, these presumed GABAergic neurons had the following electrophysiological properties that are clearly different from those in 5-HT neurons: the action potential duration was 1.19 ± 0.11 ms at the base, the action potential amplitude was 76.4 ± 2.3 mV, and the input resistance was 185.4 ± 8.5 MΩ (n = 10 cells). These putative GABAergic neurons also had deep fast afterhyperpolarization (fAHP) (Figure 1D–F). It is equally important to note that our 5-HT neurons were hyperpolarized by 5-HT. These characteristics are consistent with electrophysiological parameters for dorsal raphe 5-HT and GABAergic neurons in the literature [72,73,74,75,76], indicating that under our recording conditions, 5-HT neurons can be identified based on the whole-cell electrophysiological properties.

### 3.2. GABAergic Inhibition of DR 5-HT Cell Firing

We first determined if IPSPs inhibit the spiking activity of 5-HT neurons in our mouse brain slices, under the condition where fast ionotropic glutamate receptors were blocked by 10 μM CNQX and 20 μM D-APV. As shown in Figure 2, local electrical stimulation evoked typical hyperpolarizing IPSPs and these IPSPs clearly inhibited the generation of spontaneous action potentials in DR 5-HT neurons (n = 6). These data indicate that regulation of the GABAergic inputs may exert an important influence on 5-HT neuron activity, providing functional relevance for the experiments described below.

### 3.3. 5-HT Inhibition of GABAergic Inputs to DR 5-HT Neurons

**Figure 3:**
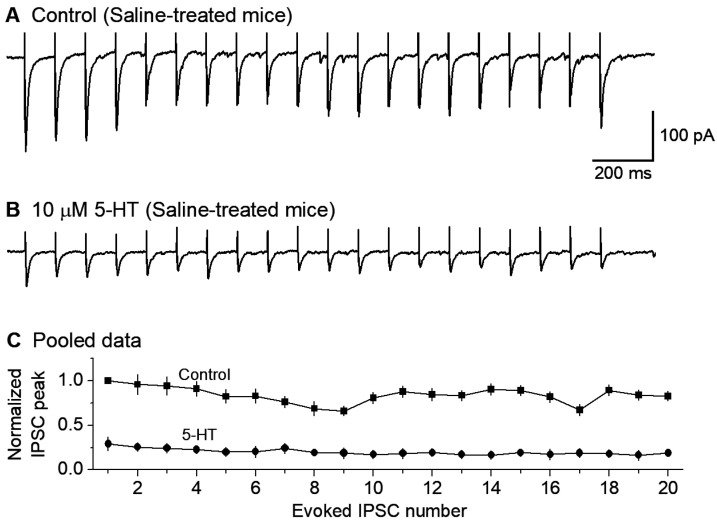
Bath-applied 5-HT substantially reduced the eIPSC amplitude in DRN 5-HT neurons in saline-treated control mice. (**A**,**B**) an example recording of a train of 20 10-Hz stimuli evoking 20 IPSCs under a basal condition (**A**) and during the application of 10 μM 5-HT (**B**). The interval between each stimulation train was 30 s. The sharp upward signals in (**A**,**B**) are stimulus artifacts. The cells were voltage-clamped at −70 mV and a 135 KCl-based intracellular solution was used, leading to inward GABA_A_ receptor-mediated eIPSCs. (**C**) Pooled data to show the 5-HT-induced reduction in eIPSC amplitude; each IPSC peak was normalized to the first IPSC peak under the control condition for each neuron. The reduction is obvious; *p* < 0.001, according to the K-S test, using data from 6 mice.

In the presence of 10 μM CNQX and 20 μM D-APV for blocking fast ionotropic glutamate receptors, we recorded local extracellular stimulation-evoked inhibitory postsynaptic currents (evoked IPSC or eIPSC) in 5-HT neurons voltage-clamped at −70 mV. To observe the repetitive GABA release properties of the afferent axon terminals, we used a train stimulation with an intra-train stimulation frequency of 10 Hz, a common spiking frequency of the neurons projecting to the raphe. At this frequency, the axon terminal can sustain the IPSC amplitude or GABA release--after a moderate depression during the first several IPSCs (Figure 3A). To examine the potential effects of 5-HT on GABAergic inputs to raphe 5-HT neurons in normal mice, we first electrically evoked this train of 20 repetitive eIPSCs. After a stable baseline recording, bath application of 10 μM 5-HT reduced the peak amplitude of each eIPSC in the train by about 70% (n = 6 neurons; Figure 3B,C).

### 3.4. GirK Channel Blocker Tertiapin-Q Prevents 5-HT Inhibition of GABAergic Inputs to 5-HT Neurons

**Figure 4:**
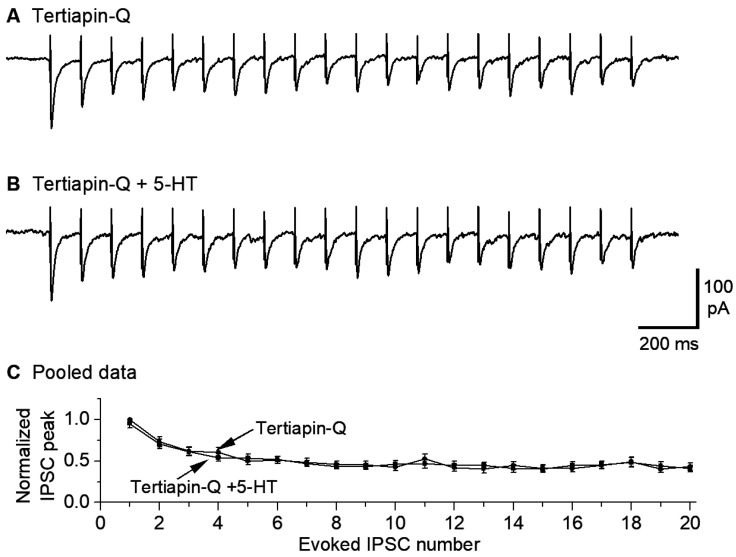
GirK channel blocker tertiapin-Q prevents 5-HT inhibition of eIPSCs in saline treated mice. A train of 20 10-Hz stimuli evoked 20 IPSCs in the presence of 1 μM tertiapin-Q only (**A**) and 1 μM tertiapin-Q and 10 μM 5-HT (**B**). The sharp upward signals in (**A**,**B**) are stimulus artifacts. The cells were voltage-clamped at −70 mV and a 135 KCl-based intracellular solution was used. For quantitative comparison (**C**), the amplitude of all evoked IPSCs in (**A**,**B**) was normalized to the first IPSC under control (i.e., 1 μM tertiapin-Q only) for each neuron; there was obviously no difference. *p* = 0.82, according to the K-S test, using data from 6 mice.

Our observation that 5-HT reduced the amplitude of repetitive eIPSCs globally suggested that 5-HT, probably via 5-HT1B receptor activation, may reduce the general excitability and hence transmitter release of the GABAergic axon terminals synapsing onto the 5-HT neurons. Specifically, 5-HT may activate GirK channels expressed in the GABAergic afferent axon terminals; recent anatomical and functional studies support this possibility in both the raphe and other brain areas [77,78,79,80]. In raphe 5-HT neurons, somatodendritic 5-HT1A couples to GirK channels that cause hyperpolarization and hence inhibition of spike firing, accompanied by decreased whole-cell input resistance and excitability. Thus, we reasoned that GirK channels expressed on raphe afferent axon terminals may reduce axonal excitability and hence cause a general decrease in GABA release. To test this possibility, we used tertiapin-Q, which is a known selective inhibitor of GirK channels [52,80,81]. As shown in Figure 4, in the presence of 1 μM tertiapin-Q, bath application of 10 μM 5-HT did not reduce eIPSCs (n = 6). These results indicate that blockade of GirK channels is a key mechanism for 5-HT to inhibit the GABAergic inputs to and GABA_A_R IPSCs in raphe 5-HT neurons.

### 3.5. Chronic Fluoxetine Treatment Downregulates 5-HT Inhibition of GABAergic Inputs to 5-HT Neurons

Chronic fluoxetine treatment is known to downregulate or desensitize 5-HT1A autoreceptors on the somatodendritic areas and axon terminals of raphe 5-HT neurons (via decreased receptor expression, receptor-G_i/o_ coupling, and/or downregulation of downstream signaling pathway). A similar downregulation of 5-HT1B receptors on non-5-HT neuron axon terminals is entirely possible but has not been reported. We hypothesized that chronic fluoxetine may induce desensitization or downregulation of presynaptic 5-HT1B inhibition of IPSCs in raphe 5-HT neurons.

**Figure 5:**
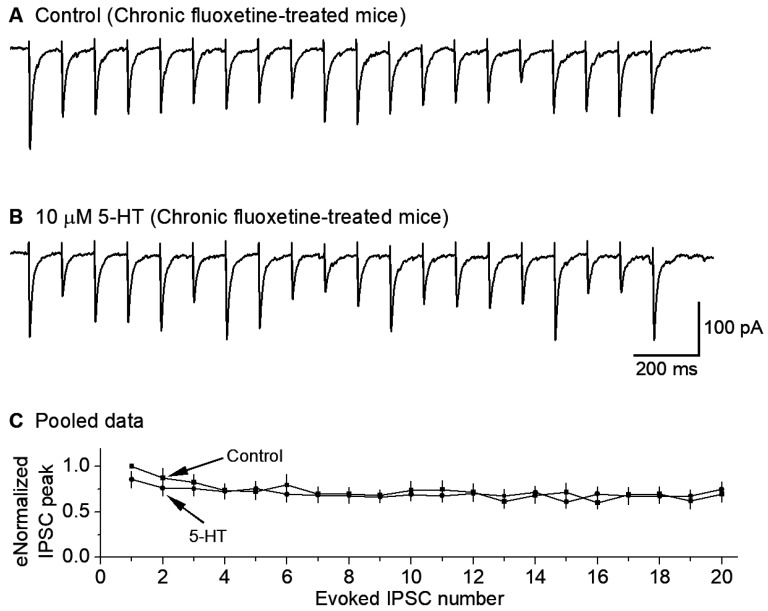
Chronic fluoxetine treatment downregulates 5-HT inhibition of GABA inputs to 5-HT neurons. A train of 20 10-Hz stimuli evoked 20 IPSCs under the basal condition (**A**) and during 10 μM 5-HT application (**B**). The cells were voltage-clamped at −70 mV and a 135 KCl-based intracellular solution was used. For quantitative comparison (**C**), each eIPSC peak was normalized to the first IPSC peak under the control condition for each neuron. *p* = 0.56, according to the K-S test, using data from 9 mice.

To test this hypothesis, we treated the mice for ≥14 days with fluoxetine (10 mg/kg per day), with a saline injection as the control. Then, we examined the effects of bath-applied 5-HT (10 μM) on the GABAergic IPSCs in raphe 5-HT neurons in fluoxetine-treated mice. We found that 10 μM 5-HT decreased the 10 Hz stimulus-train-evoked eIPSC amplitude only minimally (n = 9) (Figure 5A–C). These results indicate that chronic treatment with fluoxetine downregulates or reduces the 5-HT inhibition of GABAergic inputs to 5-HT neurons.

### 3.6. Chronic Fluoxetine Treatment Downregulates 5-HT Neuron Autoinhibition

**Figure 6:**
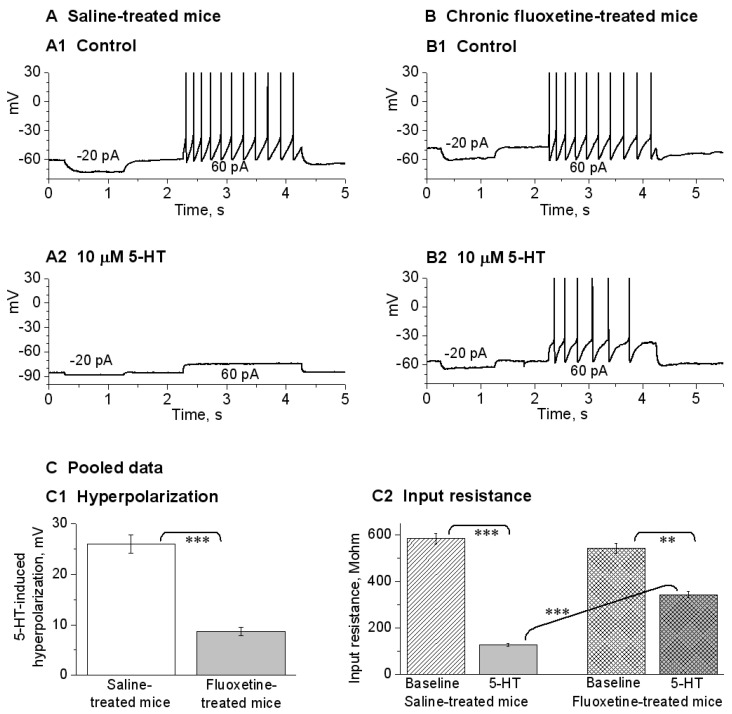
Chronic fluoxetine treatment renders DRN 5-HT neurons resistant to 5-HT autoinhibition by substantially down-regulating 5-HT autoinhibition of DR 5-HT neurons. **A**(**A1**,**A2**) In brain slices from saline-treated control mice, bath-application of 10 μM 5-HT caused large (26 mV) hyperpolarization, a large decrease in input resistance, and cessation of evoked spike firing. **B**(**B1**,**B2**) In brain slices from fluoxetine-treated mice, bath-application of 10 μM 5-HT caused much smaller (8.7 mV) hyperpolarization, a smaller decrease in input resistance, and a modest decrease in evoked spike firing. In (**A**,**B**), action potential peaks are truncated for display. **C**(**C1**,**C2**) Pooled data. The effects are obvious: ***, *p* < 0.001; **, *p* < 0.01; unpaired *t*-test for (**C1**) and one-way ANOVA for **C2**. The data are from 8 saline-treated mice and 8 fluoxetine-treated mice.

Chronic fluoxetine treatment has been shown to downregulate 5-HT1A receptors and remove the initial inhibition of 5-HT neuron firing (recorded extracellularly) [61]. Here, we used whole-cell recording to directly determine if chronic fluoxetine treatment reduces 5-HT-activated GirK current-induced hyperpolarization and the associated low excitability in raphe 5-HT neurons. We found that in saline-treated mice, bath application of 10 μM 5-HT induced robust hyperpolarization in raphe 5-HT neurons (−26.03 ± 1.82 mV, n = 8; Figure 6(A1,A2,C1)); this hyperpolarization was accompanied by a substantial decrease in input resistance (584.05 ± 23.14 MΩ under control, 128.17 ± 5.65 MΩ under 5-HT, n = 8; Figure 6(A1,A2,C2)). Further, current injection-evoked spike firing was severely inhibited (10.7 ± 0.6 spikes under control, 0 spike under 5-HT, n = 8; Figure 6(A1,A2)). These results are fully consistent with 5-HT activating 5-HT1ARs and opening GirK channels.

In mice treated with fluoxetine for 14 days, the responses to 5-HT were much smaller. Under an identical recording condition, bath application of 10 μM 5-HT induced much smaller hyperpolarization in raphe 5-HT neurons (−8.7 ± 0.9 mV, n = 8; Figure 6(B1,B2,C1)); this smaller hyperpolarization was accompanied by a smaller decrease in input resistance (542.04 ± 20.20 MΩ under control, 343.91 ± 14.26 MΩ under 5-HT, n = 8; Figure 6(B1,B2,C2)); further, current injection-evoked spike firing was only moderately reduced (10.5 ± 0.5 spikes under control, 7.5±0.5 spike under 5-HT, n = 8; Figure 6(B1,B2)). These results indicate that in chronically fluoxetine-treated mice, 5-HT/5-HT1ARs-induced GirK current in raphe 5-HT neurons is reduced. We also need to note here that although 10 μM 5-HT still inhibited DRN 5-HT neurons in brain slices from chronic fluoxetine-treated mice, the endogenous extracellular 5-HT level that probably can substantially inhibit 5-HT neurons in untreated or saline-treated animals is more likely around 1 μM, but it can no longer significantly inhibit these 5-HT neurons in SSRI-treated animals. This level needs to be determined in future studies.

## 4. Discussion

**Figure 7:**
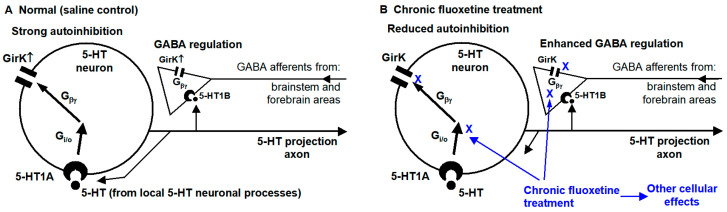
Summary diagram. In normal and saline-treated animals (**A**), 5-HT causes autoinhibition by activating somatic 5-HT1A receptors, leading to the release of the Gβγ subunit from the Gi/o protein and opening GirK channels. 5-HT may activate 5-HT1B receptors in GABA afferents and decreases GABA release, which may be mediated by activating GirK channels on axon terminals. These two effects were diminished in animals receiving chronic fluoxetine treatment (**B**).

The main findings of the present study are that (1) 5-HT, probably via 5-HT1B activating presynaptic GirK channels, reduces GABAergic regulatory inputs to raphe 5-HT neurons, and that (2) chronic treatment with the antidepressant fluoxetine downregulates this 5-HT inhibition of GABAergic inputs and also 5-HT-activated somatodendritic GirK current-mediated autoinhibition (see the summary diagram in Figure 7). Below, we will discuss these results.

### 4.1. Chronic Fluoxetine Treatment Enhances GABAergic Inhibitory Influence on Dorsal Raphe 5-HT Neurons by Downregulating Presynaptic 5-HT Inhibition

In this study, first, we demonstrated that IPSPs effectively inhibit the spike firing of DRN 5-HT neurons, partly due to their high input resistance such that even a small inhibitory synaptic input can cause significant hyperpolarization and hence inhibition of spike activity. These results are useful and expand prior studies reporting that DRN 5-HT neurons receive inhibitory synaptic inputs but do not show explicitly that these IPSPs inhibit spike firing in DRN 5-HT neurons [42,82,83,84].

Second, our data indicate that 5-HT reduced the GABAergic inputs to DRN 5-HT neurons, probably by activating presynaptic 5-HT1B receptors on GABAergic afferent terminals synapsing on 5-HT neurons based on the established observation that 5-HT1B receptors are commonly on axon terminals [42,71,82,85,86,87,88], although in the present study we did not use 5-HT1B receptor-selective ligands to test this possibility. This mechanism serves to reduce the inhibitory influence of local GABAergic neurons and external GABAergic centers on DRN 5-HT neurons; the sources of these GABAergic afferents include the hypothalamus, lateral habenula nucleus, RMTg, lateral preoptic area, pontine ventral periaqueductal gray, VTA and SNr [32,33,34,35,36,38,39,41,89]. This mechanism may contribute to the overall regulation of 5-HT neuron spiking activity that matches the animal’s mental and behavioral status and needs.

Third, our data indicate that pretreatment with the GirK channel inhibitor tertiapin-Q prevented the inhibitory effect of 5-HT on the IPSCs. Our interpretation is as follows. 5-HT activates presynaptic 5-HT1 receptors (5-HT1B and/or 5-HT1A) that in turn activate GirK channels expressed at the axon terminals, thus reducing axon terminal excitability, leading to fewer spikes, less Ca influx, and less GABA release. GirK channels have been reported to be expressed at axon terminals in multiple brain areas and also at afferent axon terminals in the DRN [77,78,79,80,90]. This is also consistent with the report that presynaptic DA receptors reduce GABA release onto midbrain DA neurons by activating presynaptic GirK channels [91].

Finally, we found that this presynaptic 5-HT inhibition was downregulated after 2-week chronic fluoxetine treatment. Previous studies have established that chronic antidepressant treatment desensitizes 5-HT1 autoreceptors on 5-HT axon terminals inhibiting 5-HT release, and this downregulation temporally coincides with the onset of antidepressant effects in animal models [54,92,93,94,95]. Our present study suggests that chronic SSRI treatment and hence extracellular 5-HT increase may affect 5-HT1B heteroreceptors. Thus, it appears that chronic exposure to high levels of extracellular 5-HT can desensitize the function and/or decrease the cell surface or de novo expression of both 5-HT1B autoreceptors and heteroreceptors. The reduced 5-HT inhibition indicates that after chronic fluoxetine treatment, 5-HT neurons can be more effectively influenced by outside GABAergic neurons in the hypothalamus, SNr, VTA, and other brain areas including the cerebral cortex and brainstem [38].

We also need to note here that literature data indicate that 5-HT1B receptors are probably expressed in GABAergic neurons in the raphe and on the axon collaterals of raphe 5-HT neurons; these 5-HT1B receptors can inhibit GABAergic and 5-HT release, and their functional roles in normal animal physiology and depression pathogenesis are being investigated but have not been established [42,93,95,96,97].

### 4.2. Chronic Antidepressant Treatment Renders DRN 5-HT Neurons Resistant to 5-HT Autoinhibition by Downregulating 5-HT Inhibition of the Intrinsic Excitability

We found that 2-week daily fluoxetine treatment substantially reduced 5-HT-induced autoinhibition of DRN 5-HT neurons monitored by whole-cell patch clamp recording. Our present results are consistent with and expand the literature data in the field. In their pioneering study using in vivo extracellular spike recording, Blier & De Montigny (1983) [57] found that ip injection of the SSRI zimelidine initially autoinhibited the extracellularly recorded spontaneous spike firing in DRN 5-HT in rats; however, following repeated daily treatment with this SSRI, the autoinhibition gradually declined and eventually disappeared after 2 weeks of treatment, demonstrating that chronic SSRI treatment that induced a chronic increase in the extracellular 5-HT level can desensitize or downregulate 5-HT autoinhibition. In a more detailed study with three doses of fluoxetine (5, 10 and 20 mg/kg per day) combining extracellular spike recording, Czachura & Rasmussen (2000) [61] confirmed that fluoxetine-induced DRN 5-HT neuron autoinhibition, although strong within the first 3 days of fluoxetine treatment, disappeared after 14 days of daily fluoxetine treatment.

The present study provides complementary whole-cell recording data that add new and useful information about the neuroplastic changes in input resistance, intrinsic excitability, and GirK current after chronic fluoxetine treatment. These important details could not be recorded in prior in vivo studies recording extracellular spikes; specifically, our data show that after 2 weeks of daily IP 10 mg/kg fluoxetine treatment, DRN 5-HT neurons became more resistant to 5-HT autoinhibition by producing a much smaller Kir current, much smaller hyperpolarization, and a much smaller decrease in input resistance. These new whole-cell data are consistent with and solidify prior extracellular spike data. Hence, in the presence of fluoxetine or another SSRI type antidepressant, 5-HT neurons can maintain their spiking activity (~1 Hz) and release 5-HT in the projection areas while the SSRI blocks reuptake and increases the extracellular 5-HT level, the intended pharmacological effect that is believed to underlie antidepressant therapeutic efficacy.

Literature evidence indicates that the following chain of events may underlie the chronic fluoxetine-induced downregulation of 5-HT neuron autoinhibition: fluoxetine blocks SERT-mediated 5-HT reuptake and thus increases the extracellular 5-HT level. A chronic increase in the extracellular 5-HT level can desensitize the 5-HT1A and 5-HT1B inhibitory autoreceptors in 5-HT neuron cell bodies and their axon terminals via diminished receptor-G-protein coupling [55,94,98,99,100,101], receptor internalization [45], or a reduction in de novo receptor gene expression [17,92,93]. These molecular events ultimately downregulate 5-HT autoinhibition and lead to sustained increases in extracellular 5-HT and thus contribute to the antidepressant effect [16,54]. The situation with presynaptic 5-HT1B receptors on non-5-HT neurons after SSRI treatment is understudied and less clear. Our study suggests that the downregulating mechanisms may be similar to those of 5-HT1A and 5-HT1B autoreceptors.

### 4.3. Limitations and Alternative Interpretation

Besides the presynaptic 5-HT1B mechanism described above, we recognize that the postsynaptic 5-HT1A receptor-activated GirK channels may contribute to 5-HT-induced reduction in evoked IPSCs in 5-HT neurons, although it has been reported that the 5-HT1A agonism did not alter the miniature IPSC amplitude in DRN 5-HT neurons recorded with a K-based intracellular solution [42], arguing against the possibility of a lower input resistance contributing to lower eIPSC amplitude. To fully resolve this issue, future studies need to use CsCl-based intracellular solutions to block postsynaptic GirK channels. We also recognize that because our study did not definitively identify the 5-HT receptors involved, future studies will need to use selective 5-HT1A ligands and 5-HT1B ligands together with transgenic 5-HT1A knockout mice and 5-HT1B knockout mice to identify the 5-HT1 receptor subtype mediating the 5-HT effects observed here. Thus, typical for scientific progress, our present interpretation may be revised when additional data are obtained, thereby refining and advancing our understanding of the raphe 5-HT neurons.

## 5. Conclusions

As depicted in Figure 7, our first conclusion is that chronic fluoxetine downregulates 5-HT inhibition (likely mediated presynaptic 5-HT1B receptors) of GABAergic synaptic inputs to DRN 5-HT neurons; consequently, chronic SSRI-type antidepressant treatment can enable extrinsic, behaviorally important GABAergic neuron activity to more effectively and closely influence DRN 5-HT neurons such that 5-HT neuron activity and hence 5-HT release better match behavioral needs. Our second conclusion is that chronic fluoxetine treatment reduces somatic 5-HT autoreceptor (likely 5-HT1A receptor)-activated GirK channel-mediated hyperpolarization and decreases the input resistance and intrinsic excitability; consequently, chronic antidepressant treatment can render DRN 5-HT neurons resistant to 5-HT autoinhibition and lead to increased 5-HT neuron activity and 5-HT release. These cellular neuroplastic events are potentially key pharmacological mechanisms by which SSRIs exert their antidepressant and other behavioral and cognitive effects.

## Data Availability

The data presented in this study are available on request from the corresponding author. The data are not publicly available due to being a part of an ongoing study.
